# T2 formula in a highly myopic population, comparison with other methods and description of an improved approach for estimating corneal height

**DOI:** 10.1186/s12886-019-1226-7

**Published:** 2019-11-12

**Authors:** Carlos Alberto Idrobo-Robalino, Gisella Santaella, Ángela María Gutiérrez

**Affiliations:** 1 Hospital Eugenio Espejo, Avenida Gran Colombia 170403, Quito, Ecuador; 2 Escuela Superior de Oftalmología - Instituto Barraquer de América, Clínica Barraquer, Calle 100 N 18 A 51, Bogotá, Colombia

**Keywords:** T2 formula, High myopia, Corneal height estimation, Cataract surgery, Intraocular lens calculation

## Abstract

**Background:**

To determine the accuracy of the T2 formula as applied to highly myopic eyes, to compare the T2 formula to the SRK/T and Holladay 1 formulas, and to describe possible ways to improve the estimation of corneal height and prediction error in two settings, the Hadassah Hospital, Ophthalmology Department, Jerusalem, Israel and Clínica Barraquer, Bogotá, Colombia.

**Methods:**

In this retrospective case series, optical biometer measurements were taken for 63 highly myopic patients (> 25 mm) undergoing uneventful crystalline lens phacoemulsification and insertion of an acrylic intraocular lens. Prediction errors were obtained, with estimations of ±0.50 D, ± 1.00 D, and greater than ±2.00 D. A method to improve the corneal height calculation is described.

**Results:**

The SRK/T formula (mean absolute error [MAE] = 0.418; median absolute error [MedAE] = 0.352) was the most accurate, followed by the T2 (MAE = 0.435; MedAE = 0.381) and Holladay 1 (MAE = 0.455; MedAE = 0.389) formulas. Both the SRK/T and T2 formulas overestimated corneal height, but values were higher with the T2 formula. Corneal height was more precisely estimated using an alternative method that, when combined with axial length optimization, resulted in lower MAE (0.425) and MedAE (0.365) values than when applying the T2 formula alone.

**Conclusions:**

The T2 formula seems to be less accurate than the SRK/T formula in highly myopic eyes. An improved corneal height estimation method is described for the the T2 formula.

## Background

Highly myopic eyes have a long axial length (L); (> 25 mm), a deep anterior chamber depth (ACD), and a floppy capsular bag, therefore, calculating the intraocular lens (IOL) power of these eyes is challenging and often results in a postoperative hyperopic surprise. The use of partial coherence interferometry [1] together with specific formulas (e.g. Barrett Universal II [2] and Haigis [3]) are strategies to improve the IOL estimation in these cases.

The SRK/T formula is a well-known method with evidence reporting its accuracy in cases of high myopia [2]. The size of the postoperative anterior chamber and the position of the IOL are predicted by the SRK/T using the following concepts: 1) The corneal height (H), is a model in which the cornea is regarded as a section of a sphere, the base of which forms a plane at the level of the anterior iris, therefore H can be defined as the distance from the anterior surface of the iris to the central cornea, in the SRK/T paper measures dealing with this value included the corneal thickness [4]. 2) Corrected Axial Length (LCOR): The SRK/T assumes that the vitreous chamber size undergoes a greater elongation than the anterior segment, As a result, this formula applies a correction factor in eyes longer than 24.2 mm of axial length which allows for a more accurate estimation of ACD in the long eye, this adjustment is used as part of the corneal height (H) estimation [4]. 3) Offset: Below the iris, and with the IOL in position, the offset is the distance from the iris plane to the optical plane of the IOL.

In spite of the advantages of the SRK/T, authors like Haigis [5] observed that it was not as effective in certain situations. For instance, in the calculation of the ACD, when the corneal width is two times greater than the corneal radius, the formula attempts to calculate the square root of a negative number, a phenomenon termed “imaginary ACD.” This event is controlled by changing the described negative value to zero, an adjustment that only represents a partial solution, and that induces a non-physiological behavior, called the “SRK/T cusp.” [6]

The T2 formula was developed as a method which would tackle the pitfalls of the SRK/T, its authors describe two sources of error for the original formula [1]: LCOR reversal, where LCOR progressively decreases as AL values exceed 36.2 mm; and [2] the SRK/T cusp, corrected by replacing steps 2 to 4 in the original SRK/T formula with a regression formula for corneal height [6] (from now on called H2). The T2 formula corrects estimation errors of H but its benefits are not as evident as expected in long eyes [7, 8].

An important feature of the design of the T2 equation is that it uses L without any correction (avoiding the LCOR step from the SRK/T), and the keratometry. Interestingly, a second formula for the corneal height was developed in the original report on the T2 formula, which does include LCOR (termed H2.2 herein) and which will be of special interest in this paper. Appendix 2 presents all aforementioned equations.

The Holladay 1 formula has also been successfully used in normal and myopic eyes [4], and it has been included in the present study for comparison purposes, due to its similar design to the SRK/T.

The present investigation compared the outcomes of the SRK/T, T2, and Holladay 1 formulas in highly myopic eyes. In addition, it analyzed the SRK/T and T2 formulas in order to find options to improve the prediction of H in very long eyes.

## Methods

An observational retrospective chart review was performed. This review included 63 highly myopic patients (> 25.00 mm), who underwent uneventful crystalline lens phacoemulsification and IOL insertion at one of two clinics: the Hadassah Ein Keren Ophthalmology Clinic, Jerusalem, Israel (39 cases from June 2012 to January 2014) and the Clínica Barraquer, Bogotá, Colombia (24 cases from February 2013 to November 2015). Institutional review board approval was obtained, and all methods adhered to the Helsinki Declaration. Inclusion criteria were as follows: highly myopic eyes (L > 25 mm), Alcon Acrisoft® SN60WF acrylic IOL in-the-bag implants, and postoperative visual acuity ≥20/40. Exclusion criteria were as follows: absent or inadequate optic biometry and/or conditions affecting best corrected visual acuity (e.g. choroidal neovascularization, optic atrophy, etc.). Myopic retinal degeneration and glaucoma were reasons for exclusion only if severely impairing.

The measured variables were as follows: L and keratometry (measured with Carl Zeiss IOL Master® Optical Biometer); preoperative and postoperative best corrected visual acuity (measured with ETDRS chart and converted to LogMAR notation using an online tool [http://www.myvisiontest.com/logmar.php]; postoperative refraction (measured at minimum one month post-operation). The Holladay 1, SRK/T, and T2 formulas were included for assessments. The applied A-constant and Surgeon Factor were respectively 119.0 and 1.84 (based on recommendations from the User Group for Laser Interference Biometry) [9].

The IOL powers for predicted refraction and emmetropia were estimated. Prediction error was defined as the difference between the refractive error calculated by the formula and the stable postoperative refraction. Calculations were performed using verified formulas developed by Dr. Richard Sheard (Microsoft Excel Functions Add-In Version 4.2).

The estimation of errors was as follows: Mean Error (ME) was made equal to zero by changing the lens factor individually for each formula, this was achieved using the Excel software’s Data/What If Analysis/Goal Seek function [10], after this procedure, constants obtained were: A constant for SRK/T: 119.21; A constant for T2: 119.23; A Constant for T2 formula including H2.2 and Wang’s AL optimization (described below): 118.63; Surgeon Factor for Holladay 1: 2.27.

After the mean errors were zeroed out, all negative values were converted to positive and the mean absolute error (MAE) was reported for each formula. Then, Median Absolute Error (MedAE) was calculated. Standard, minimum and maximum errors were estimated, together with the percentage of eyes with prediction errors ≤ ±0.50 diopter (D), ≤ ± 1.00 D, and ≤ ±2.00 D [10].

The overall sample was analyzed to avoid subgroup bias. H was calculated using steps 2 to 4 of the SRK/T formula [4] (termed hereafter as HSRK/T), and two equations described by Sheard et al. [6] (H2 and H2.2). Correlative analyses were performed using commercially available software (Excel 2013, SPSS v.17.0).

Eyes with previous corneal surgery or corneal diseases, and preoperative pathologic changes affecting central vision were excluded. Foveal and perifoveal integrity together with confirmation of stability of any condition were required before inclusion in the sample for analysis.

## Results

### Sample description

The demographics of each sample group (i.e. 39 cases from Hadassah Ein Keren Hospital and 24 from Barraquer Clinic) are detailed in Table 1.

**Table 1:** Demographics of the two studied groups

Group	Ethnicity	Mean Age	Gender	Laterality	N
Hadassah Ein Keren Hospital, Jerusalem, Israel	Jewish; Arabic	68.67 yo, SD ± 10.25 Min: 43 Max: 85	Male: 43.85% Female: 56.41%	Right: 58.97% Left: 41.02%	39
Clínica Barraquer, Bogotá-Colombia	Latin American - Hispanic	60.41 yo, SD ± 12.14 Min: 37 Max : 81	Male: 41.66 % Female: 58.33%	Right: 66.6 % Left: 33.3 %	24

*yo* years old, *SD* Standard Deviation, *Min* Minimum, *Max* Maximum, *n* Number of eyes Studies

The pre and post-operative statuses of the assessed variables are summarized in Table 2.

**Table 2:** Variables included in the present study

Variable	Mean	Standard Deviation	Minimum	Maximum
PreOp VA (Logmar)	0.494	0.346	0.041	1.477
PostOp VA (Logmar)	0.101	0.1043	0	0.301
Flat K	42.99 D	1.61906832 D	39.38 D	46.81 D
Steep K	44.09 D	1.76891495 D	40.23 D	48.5 D
Mean K	43.54 D	1.62941283 D	40.08 D	47.2 D
L	26.94 mm	1.107 mm	25.22 mm	30.08 mm

*PreOp* Preoperative, *PostOp* Postoperative, *VA* Visual acuity, *K* Keratometry, *L* Axial length; *n* = 63

The target preoperative refraction had a mean of − 1.171 (Min − 5 Max: 0.68, SD 1.330). Whereas the postoperative refraction had a mean Sphere of − 0.783 (Min − 4.25; Max:1.5; SD 1.382) and a mean Cylinder of - 0.900 (Min − 4 Max: 0, SD 0.745).

Preoperative pathology was found in eight out of 63 eyes (12.69%): one case of uveitis (1.59%), one case of temporary diplopia (1.59%), one case of pseudo exfoliation syndrome (1.59%), one case with peripheral lesions requiring laser treatment (1.59%), and one case of extrafoveal choroidal neovascularization (1.59%). Three patients presented with atrophic macular changes outside the fovea (4.76%). Any pathology found was confirmed to be stable and not affecting visual acuity before cataract surgery took place, these cases were allowed in the analysis group provided that none of the changes was found to affect visual acuity.

### Ranking of formulas

**Fig 1:**
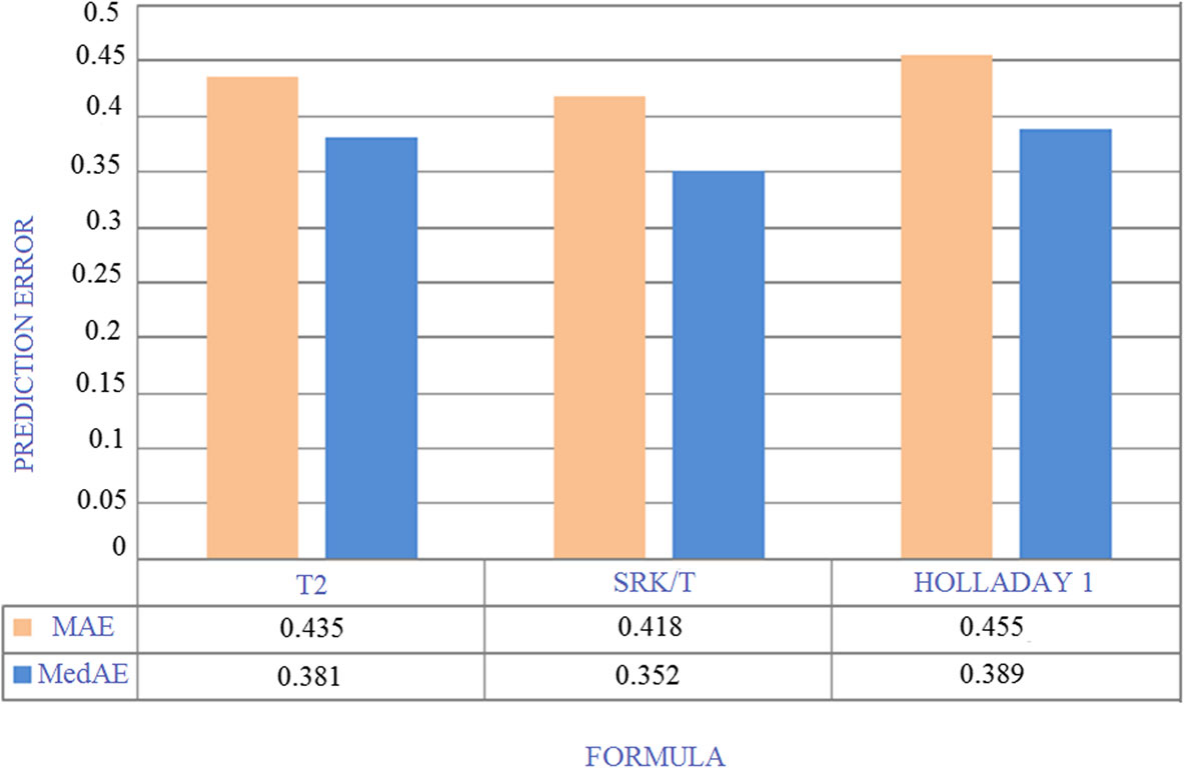
Median and Mean Absolute Error of the T2, SRK/T and Holladay 1 formulas Abbreviations: MAE: Mean Absolute Error; MedAE: Median Absolute Error; T2: T2 formula; SRK/T: SRK/T formula, *n*=63

Of the tested equations, the most accurate was the SRK/T formula (MedAE = 0.352), followed by T2 (MedAE = 0.381) and Holladay 1 (MedAE = 0.389) formulas (Table 3, Fig. 1). Lin’s correlation [11] factor was used to analyze the MedAE of the three methods (Table 4).

**Table 3:** Summary of the prediction error in the present study

Formula	MAE	Standard Deviation	Minimum	Maximum	MedAE	≤ ± 0.50 D	≤ ± 1.0 D	Sum of errors ≤ ± 0.50 D + ≤ ± 1.0 D	> 2.00D
SRK/T	0.418	0.327	0.003	1.359	0.352	71.42%	20.63%	92.05%	7.93%
Holladay1	0.455	0.314	0.037	1.404	0.389	61.90%	31.74%	93.64%	6.35%
T2	0.435	0.328	0.014	1.389	0.381	69.84%	22.22%	92.06%	7.94%

*MAE* Mean absolute error, *MedAE* Median absolute error, *T2* T2 formula, *n* = 63

**Table 4:** Lin’s correlation coefficient of the median absolute error of the methods used in the present study

	T2	HOLLADAY 1
SRK/T	*ρ*_*c*_ *= 0.9829* 95% CI = 0.9720 to 0.9896	*ρ*_*c*_ = 0.9537 95% CI = 0.9253 to 0.9715
T2		*ρ*_*c*_ = 0.9575 95% CI = 0.9311 to 0.9739

*ρ*_*c*_: Lin’s concordance correlation coefficient, 95% CI: 95% confidence interval. *n* = 63

A substantial correlation was found between the T2 and SRK/T formulas. Correlations between the SRK/T and Holladay 1 formulas and between the Holladay 1 and T2 formulas were also substantial, but with only moderate lower limits of the confidence intervals.

### Analysis of calculation methods

Since the main difference between the T2 and SRK/T formulas is the estimation of H, the behaviors of L and keratometry were analyzed respect to Corneal Height.

L is used without any modification in H2, while an adjusted L (LCOR) is required by the HSRK/T formula. A correlative analysis was performed between both H-calculation methods and L, with the results being a very low correlation between HSRK/T and L (Table 5) but a strong positive correlation between H2 and L (*r* = 0.808; *p* < 0.05).

**Table 5:** Correlation between different methods of Corneal Height estimation and associated variables

	Axial Length	Mean Keratometry
HSRKT	*r* = 0.224	*r* = 0.805
	*p* = 0.078	*p* < 0.01
H2	*r* = 0.808	*r* = 0.265
	*p* < 0.01	*p* < 0.05
H2.2	*r* = 0.425	*r* = 0.695
	*p* < 0.01	*p* < 0.01

HSRK/T: Corneal height estimation using SRK/T, H2: Corneal height estimation using T2, H2.2: Corneal height estimation using the alternative T2 formula

This finding is important for the following reasons [1]: it suggests that L has a strong effect on the estimation of H calculated with the method included in the T2 formula [2]; it might explain the higher MedAE seen in highly myopic eyes with the T2 formula; and [3] it indicates that LCOR may be why L has less impact when H is estimated with the SRK/T approach.

In summary, modifying the calculation of H in the T2 formula improves its accuracy, resulting in a lower MedAE in eyes with normal L. However, the benefit of this adjustment seems to be lost in longer eyes, probably due to the effect of L on the estimation of H. On the other hand, the SRK/T formula seems to be less affected by an extreme L, which could be associated with the inclusion of LCOR in its design.

The second variable needed to calculate H is the keratometry. The average keratometry was found to have a strong positive relationship with HSRK/T (*r* = 0.805, *p* < 0.05), but a negligible correlation with H2 (*r* = 0.265, *p* < 0.05).

### Improvement options

#### Corneal height (H)

The performed analyses suggested that the presence of LCOR reduces the impact of extreme AL values in the estimation of H. Therefore, including the corrected AL in the T2 formula might improve its behavior in long eyes. Therefore, a formula which might both, solve the SRK/T cusp problem and include LCOR was needed. The easiest way to complete this task was using the second regression formula described by Sheard et al. in the original paper on the T2 formula. This second regression formula was excluded from the final T2 method because of its slightly lower correlation [6]. In the present study, this formula is named H2.2 and is calculated as follows:

H2.2 =  − 11.980 +   0.38626 × LCOR + 0.14177 × K

**Fig 2:**
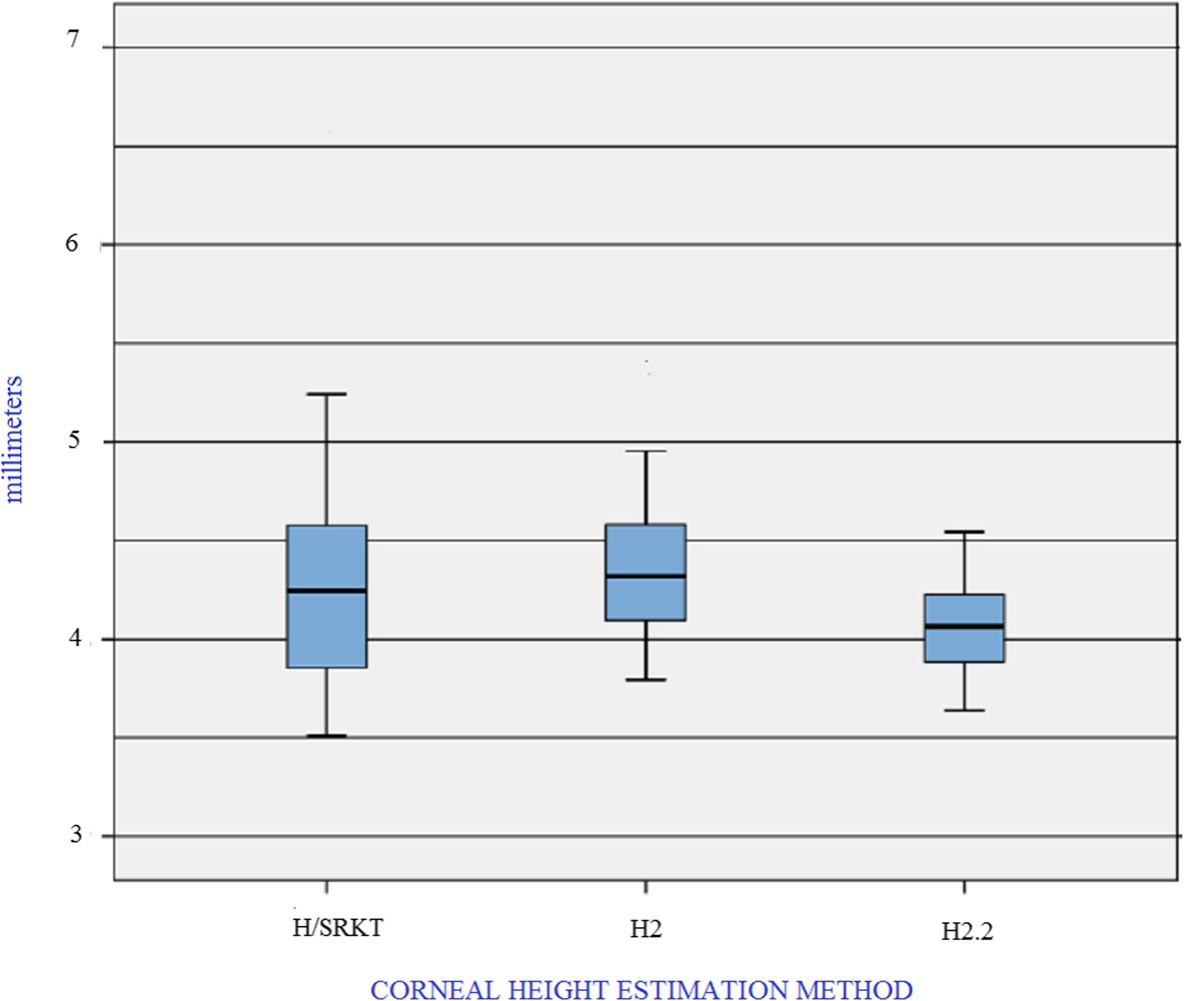
Box plot of Corneal Height estimations using SRK/T, T2 and the alternative Corneal Height method described. Abbreviations: HSRK/T: Corneal Height estimation using steps 2 to 4 of the SRK/T formula; H2: Corneal height estimation using equation number 1 for H described by Sheard et al. [6] and programed in the T2 formula. H2.2: Corneal height estimation using equation number 2 for H described by Sheard et al. [6] and applied in the present work. *n* = 63

Estimations of H using the H2.2 formulas were compared with results obtained using the HSRK/T and H2 formulas (Fig. 2, Table 6). The H2.2 method reduced the mean H value and the reported range of values.

**Table 6:** Corneal Height estimation using three methods

	Minimum	Maximum	Mean	Standard Deviation
HSRKT	3.5101	6.6086	4.2713	±0.5490
H2	3.7947	5.4057	4.3567	±0.3503
H2.2	3.6395	4.7624	4.0631	±0.23624

HSRK/T: Corneal height estimation using SRK/T, H2: Corneal height estimation using T2, H2.2: Corneal height estimation using the alternative T2 formula, *n* = 63

Statistically significant differences were found between the H2.2 and H2 formulas (*p* < 0.005), as well as between the H2.2 and HSRK/T formulas (*p* < 0.005). A moderate correlation was found between H2.2 and average keratometry (*r* = 0.695, *p* < 0.05), and a low correlation was found between L and H2.2 (*r* = 0.425, *p* < 0.05).

These results suggest that the H2.2 formula might improve H estimations, reducing the mean H, the range of extreme values, and the influence of very high keratometry and L values.

When H2.2 was used to estimate IOL, the MAE and MedAE were respectively 0.433 and 0.3815 (Table 7).

**Table 7:** Prediction error applying T2 with the alternative corneal height estimation method and optimization of axial length

Formula	MAE	Standard Deviation	Minimum	Maximum	MedAE	≤ ± 0.50 D	≤ ± 1.0 D	Sum of errors ≤ ± 0.50 D + ≤ ± 1.0 D	> 2.00D
T2 using H2.2 alone	0.433	±0,0117	0.0032	1.3856	0.3816	69.84%	22.22%	92.06	7.93%
T2 using H2.2 and optimized L	0.425	±0.3318	0.0025	1.382	0.3648	68.25%	23.81%	92.06	0%

H2.2 = Corneal height estimation according to the alternative T2 formula, Optimized L: Adjustment of L according to Wang L et al. [12] *n* = 63

While these results are only slightly better than T2 formula, a better estimation of H in highly myopic patients is obtained.

#### Optimized axial length

An additional approach to improve results of the T2 formula in highly myopic eyes is to optimize axial length. Since H2.2 includes LCOR, the method described by Wang L et al. [12] for the SRK/T formula can be used directly. When this approach was tested, the MedAE and MAE were even lower than obtained with H2.2 alone (Table 7).

## Discussion

The accuracy of the SRK/T formula in highly myopic patients has long been established [2, 7, 13], in spite of this, flaws estimating H have been described [5, 6]. The T2 formula, developed by Sheard et al. [6], improves H prediction and significantly reduces the prediction error in normal eyes. It could, therefore, be assumed that the T2 formula would perform better than the SRK/T formula among highly myopic patients, but the present investigation found that SRK/T formula could still be a better choice.

The SRK/T approach for estimating H utilizes keratometry and L, The axial length estimation is corrected using LCOR when it is higher than 24.2 mm [4]. The resulting H value in highly myopic patients includes errors such as the H cusp and LCOR reversal [6], both of which result in a far greater H estimation than what could be considered normal, even for myopic patients. This is evident when studies of corneal height measurement in vivo are considered. For instance, Dong Hyun Kim et al. [14] reported a mean H value of 3.71 ± 0.23 mm, measured by optical coherence tomography, in patients with a mean L of 28.00 mm. Another study comparing the eyes of anisometric patients reported that ACD did not differ greatly between the shorter and longer eye, even when very highly myopic patients were included. Therefore, ACD and H values in highly myopic patients do not differ extremely from the values for normal eyes. The increased L in highly myopic eyes depends mostly on the vitreous cavity and not on an extremely deep anterior chamber [15].

The T2 formula solves the H cusp problem [6], but the equation used in the original report did not include LCOR. According to the findings of the present study, LCOR might be an important factor related to the higher precision of the SRK/T formula in highly myopic eyes. In addition, the H2 equation, included in the T2 formula, resulted in a higher mean H than the method used by the SRK/T formula. This could partially explain the higher MedAE and MAE values when applying the T2 formula to highly myopic eyes.

In this regard, the solution to improve the T2 prediction error proposed in the present study includes two parts. First, since LCOR helps improve the H estimate in the SRK/T formula, this step was included in the T2 estimation of H, specifically using the second regression formula described in the report on the T2 formula [6]. The result of this change was a more precise H estimation than that obtained using either HSRK/T or the regular H2 method. The second step was to improve L estimation. This goal was accomplished by using a published L optimization equation for SRK/T [6], which resulted in lower MAE and MedAE values than those observed using T2 alone.

An issue of including LCOR in the T2 formula might be that in very long eyes (i.e. L > 36.2 mm) the LCOR reversal phenomenon appears, therefore a formula that uses the SRK/T platform together with additional solutions should assess this concern to best fit the requirements of long eyes. Methods to optimize L could be applied directly to the T2 formula or the described H2.2 method.

Other studies have tested the T2 formula in different settings (Table 8), and no definitive consensus exists regarding the accuracy of the T2 vs SRK/T formulas in long eyes. One study found better results using SRK/T [7], while another described better accuracy using T2 [8]. The results of the present study are similar to previous analyses of the SRK/T and Holladay 1 formulas [2, 7], but new information is provided in relation to calculating H. Suggestions for improving IOL calculations in highly myopic patients are provided. Despite these contributions, an important limitation of the present study is the relatively small sample size. This limitation is due to the relative infrequency of highly myopic eyes, even among very large sample pools. The inclusion of more highly myopic cases may be needed to clarify the presented observations and to develop necessary optimization formulas.

**Table 8:** Comparison of studies that include the T2 formula

	Kane, et al. (2016)	Cooke & Cooke (2016)	Sheard et al. (2010)	Present study
Total studied eyes (# of long-eyes)	3241 (77)	1454 (54)	11189 (not target of study)	63 (63)
Long eye definition	> 26.0 mm	PCI (25.97–29.44 mm) OCLR (26.02–29.51 mm)	Not target of study	> 25.0 mm
Formulas: MAE/MedAE	T2: 0.498/0.440 SRK/T: 0.484/0.419 Holladay 1: 0.586/0.441	PCI group T2: 0.319/0.269 SRK/T: 0.399/0.368 Holladay 1: 0.495/0.473 OLCR group T2: 0.293/0.251 SRK/T: 0.392/0.344 Holladay 1: 0.505/0.479	T2: MAE = 0.306 SRK/T: MAE = 0.3229	T2: 0.435/0.381 SRK/T: 0.418/0.352 Holladay 1:0.455/0.389 T2 with H2.2 and optimized L: 0.425/0.3648

Only results concerning the studied formulas are shown. *PCI* Partial coherence interferometry, *OLCR* Optical low coherence reflectometry

Calculating the IOL in highly myopic eyes is still a complicated issue, and even with modern formulas, errors still exist. This reality underscores the importance of continued investigation and improvement in this subject. The SRK/T formula is one of the most accurate for long-eyed patients with the advantage of being readily available in different settings. Therefore, improving this method remains a relevant aim, even in the presence of new generation formulas. Additionally, a more accurate estimation of H might benefit eyes with steep or irregular corneas, such as those observed after refractive surgery or in the presence of keratoconus, where the use of a value closer to normal may lower prediction errors. The fact that the most important source of error in third generation formulas is the ACD estimation [16] makes the findings of this study relevant and points to ways for physicians to improve their calculations in highly myopic patients.

## Conclusions

The T2 formula is recognized as the most precise option compared to the SRK/T and Holladay 1 formulas for the overall population (i.e. normal eyes). Nevertheless, evidence is contradictory regarding its accuracy in the highly myopic.

This paper provides evidence showing that T2 is less precise than SRK/T in the highly myopic eyes and describes a method to improve the corneal height estimation and the accuracy of the T2 formula.

A future study with more patients would be important in order to verify the findings in this paper. The addition of very long eyes, optimized constants, different intraocular lens designs and more formulas (like Olsen and Haigis) would allow for better comparison and confirmation of the effects found here.

## Appendix

Steps for calculating corneal height using the SRK/T method:

1. Corneal radius of curvature, *r* = 337.5/K.

2. Corrected axial length, LCOR:

If L ≤ 24.2 then LCOR = L

If L ≥ 24.2 then LCOR = − 3.446 + 1.716 L – 0.0237 × L^2^

3. Computed corneal width (C_w_)

$$ {\mathrm{C}}_{\mathrm{w}}=-5.40948+0.58412\times \mathrm{LCOR}+0.098\times \mathrm{K} $$

4. Corneal height (H)

$$ \mathrm{X}={\mathrm{r}}^2-\left({\mathrm{Cw}}^2/4\right) $$

$$ \mathrm{If}\ \mathrm{x}<0\ \mathrm{then}\ \mathrm{x}=0 $$

$$ \mathrm{H}=\mathrm{r}-\sqrt{\mathrm{X}} $$

5. Offset for specific intraocular lens (IOL) to be implanted

$$ \mathrm{Offset}={\mathrm{ACD}}_{\mathrm{const}}-3.336 $$

Steps in the T2 formula for calculating corneal height (H2).

$$ \mathrm{H}2=-10.326+0.32630\times \mathrm{L}+0.13533\times \mathrm{K} $$

Alternative formula for estimating T2 (H2.2).

$$ \mathrm{H}2.2=-11.980+0.38626\times \mathrm{LCOR}+0.14177\times \mathrm{K} $$

## Abbreviations

95% CI: 95% confidence interval
A: Constant used for *SRK/T*^5^
ACD: Anterior Chamber Depth
ACDconst: Constant used for anterior chamber depth in *SRK/T* formula for specific IOL/surgeon; can be computed from A-constant^5^
Cw: Corneal width computed from L and K (mm) ^5^
D: Diopters
ETDRS: Early Treatment Diabetic Retinopathy Study visual acuity test
H: Corneal Height – theoretical estimation of the distance from a plane which lies above the anterior surface of the iris and the top of the central cornea at its endothelial surface, this model regards the cornea as a dome which base lies at the anterior iris. The corneal width and the corneal curvature are employed to estimate this value
H2: Corneal Height Calculated with formula number 1, described by Sheard et al.^7^
H2.2: Corneal Height Calculated with formula number 2, described by Sheard et al. ^7^
HSRK/T: Corneal Height Calculated with steps 2 to 4 of the SRK/T formula^5,7^
IOL: Intraocular Lens
K: Keratometry. In Appendix 1 it refers exclusively to the averaged Keratometry where the abbreviation was kept in order to preserve the original description of the SRK/T^5^
L: Axial length measured using ultrasound in the original SRK/T paper^5^ and the IOL Master Biometer® (mm) in this paper
LCOR: Axial length with long eye correction; used in height formula^5^
LogMar: Logarithm of the Minimum Angle of Resolution
MAE: Mean Absolute Error
Max: Maximum
MedAE: Median Absolute Error
Min: Minimum
mm: millimeters
n: Number of eyes studied
offset: Difference between corneal height of the average eye and the ACD-constant of a given IOL^5^
OLCR: Optical low coherence reflectometry
PCI: Partial coherence interferometry
PostOp: Postoperative
PreOp: Preoperative
r: averaged corneal radius of curvature (mm)^5^
SD: Standard Deviation
SN60W: Biconvex, Aspheric Intraocular lens model by Alcon®, made of an Acrylate/Methacylate Copolymer
SRK/T: Third generation formula for intraocular lens calculation developed by Sanders, Retzlaff, and Kraff
T2: Formula developed by Sheard et al. for intraocular lens calculation based on the SRK/T^7^
T2.2 OPTAL: Calculation of introaocular lens using two improvement methods for the SRK/T formula: the H2.2 formula for corneal height^7^ and the optimized axial length by Wang et al.^13^
VA: Visual acuity
X: Mathematical estimation used as part of the calculation of the Corneal Height in the SRK/T formula
yo: years old
*ρ*_*c*_: Lin’s concordance correlation coefficient
